# Clinical Observations and Treatment Approaches for Scoliosis in Prader–Willi Syndrome

**DOI:** 10.3390/genes11030260

**Published:** 2020-02-28

**Authors:** Harold J.P. van Bosse, Merlin G. Butler

**Affiliations:** 1 Shriners Hospital for Children, 3551 North Broad Street, Philadelphia, PA 19140, USA; 2 Departments of Psychiatry & Behavioral Sciences and Pediatrics, University of Kansas Medical Center, Kansas City, KS 66160, USA; mbutler4@kumc.edu

**Keywords:** Prader–Willi syndrome, scoliosis, kyphosis, spinal deformities, junctional kyphosis, risk factors, treatment options, surgery, bracing

## Abstract

Prader–Willi syndrome (PWS) is recognized as the first example of genomic imprinting, generally due to a de novo paternal 15q11-q13 deletion. PWS is considered the most common genetic cause of marked obesity in humans. Scoliosis, kyphosis, and kyphoscoliosis are commonly seen in children and adolescents with PWS with a prevalence of spinal deformities cited between 15% to 86%. Childhood risk is 70% or higher, until skeletal maturity, with a bimodal age distribution with one peak before 4 years of age and the other nearing adolescence. As few reports are available on treating scoliosis in PWS, we described clinical observations, risk factors, therapeutic approaches and opinions regarding orthopedic care based on 20 years of clinical experience. Treatments include diligent radiographic screening, starting once a child can sit independently, ongoing physical therapy, and options for spine casting, bracing and surgery, depending on the size of the curve, and the child’s age. Similarly, there are different surgical choices including a spinal fusion at or near skeletal maturity, versus a construct that allows continued growth while controlling the curve for younger patients. A clear understanding of the risks involved in surgically treating children with PWS is important and will be discussed.

## 1. Introduction

Prader–Willi syndrome (PWS), a rare syndrome caused by errors in genomic imprinting, is considered the most common genetic cause of marked obesity in humans [1]. It occurs in about one in 15,000 to 30,000 births with an estimated 400,000 affected individuals worldwide [2]. PWS characteristics at birth and infancy include hypotonia with a poor suck reflex leading to feeding difficulties, and failure to thrive [3,4]; hypogonadism and hypogenitalism are present, associated with varying deficiencies in growth, thyroid and sex hormones; developmental and cognitive delays range between mild and severe. Small hands and feet, short stature, and mild facial dysmorphic features are recognized, especially in children lacking growth hormone treatment. Behavioral problems arise at different stages of childhood, including skin picking, temper tantrums and outbursts, obsessive compulsions and food seeking (hyperphagia). If not externally controlled, the hyperphagia, along with decreased physical activity and a low metabolic rate, can lead to obesity; the obesity itself is often life-threatening.

PWS results from loss of gene expression of the paternally derived chromosome 15q11-q13, most commonly (60%) due to a deletion of the region. The second most common cause (35%) is maternal disomy of chromosome 15 (UPD), where both copies of the chromosome are maternally inherited without a paternal copy. The remaining cases have an error in the center that controls the activity of the region’s imprinted genes or from translocations or inversions involving chromosome 15 [5]. Children and adults with the deletion form of PWS are more prone to self-injury, compulsions and lower cognition than those with maternal disomy 15 [6,7]. In comparison, those with maternal disomy 15 are at a greater risk of autistic features and psychosis during late adolescence or early adulthood [1].

Scoliosis, kyphosis, and kyphoscoliosis are commonly seen in children and adolescents with PWS. Spinal deformities are measured using the Cobb angle method, where lines are drawn along the endplates of the uppermost and lowermost vertebra contained in a curve, and the curve value is measured as the angle between these two endplate lines. A typical child of any age has virtually no measurable deviation from a straight spine. Curves under 10° are considered not to be scoliotic. Curves between 10° and 20° are closely observed, but treatment is usually withheld until a curve shows obvious progression or is larger than 20°–25°. The prevalence of spinal deformities is cited between 15% and 86% [8,9,10,11,12,13]. For example, a 2007 survey in the USA of caregivers of persons with PWS by the Prader–Willi Syndrome Association ((PWSA (USA)) found that 40% of 1603 respondents were positive for scoliosis and/or kyphosis. This is similar to studies reviewed earlier by Butler in 1990 [3] in summarizing 538 patients found in the literature prior to growth hormone treatment with 44% having scoliosis. In the PWSA (USA) survey, the female to male ratio for those with minor curves was 1.23:1, and 2.3:1 for curves requiring treatment (bracing or surgery). Females had about a 10% higher chance of developing scoliosis, but both sexes had an equal risk of curve progression. As for the PWS genetic classes, those with maternal disomy 15 appeared to have a slightly higher risk of developing scoliosis, but no PWS genetic type had a higher risk of progression. The survey also identified two peak age incidences for spine deformities, at 0–24 months, and the second at 73–96 months, indicating a bimodal age distribution. The 40% prevalence rate is probably an underestimate, as many participants were included before they reached skeletal maturity, and scoliosis was identified by caretakers’ knowledge of a spine deformity rather than on spine radiographs. In studies that used radiographs and stratified by age, an overall higher prevalence was found. Odent et al. found an overall rate of 43%, but 68% for those that reached skeletal maturity [14]. Nagai et al noted 22% of patients 5 years or younger had scoliosis, 25% for those 6–11 years, and 68% for those over 12 years of age [12]. Similarly, de Lind van Wijngaarden et al. reported a prevalence of 23% for infants, 29% for juveniles, and 12 of 15 adolescents (80%) [8]. These studies support a bimodal age pattern for scoliosis in PWS, with 23% of patients developing curves before their 4^th^ birthday, likely due to hypotonia. Relatively few patients were found in the juvenile period (2–6%), and some may have had curves missed during the infant period. Near adolescence, a second substantial curve onset occurs with approximately 45% of children. Overall, the cumulative risk of developing a spine deformity appears to be about 70%.

Patients with PWS can exhibit both the C-shape and the S-shaped curves. The C-shape is primarily a sign of the underlying hypotonia where the child will list asymmetrically to the side. As the child gains strength, they are able to compensate above and below the initial curve, thereby functional centering the head over the pelvis. This converts the curve to an S-shape. The C-shaped curve may be referred to as uncompensated while the S-shaped curves are compensated. 

Reasons for treating spine deformities in PWS are not so much related to body image; although children and adults with PWS are body-conscientious, most do not display the same self-awareness as seen in their peers. The decreased respiratory status of children and adults is the more important issue, with elements of sleep apnea (both obstructive and central), weak respiratory musculature, and nocturnal hypoventilation [15,16,17,18]. Scoliosis and/or kyphosis are often indicated as significant co-morbidities for respiratory compromise, by virtue of associated chest deformities, although conclusive proof is still lacking [10,19,20,21,22,23]. People with PWS have a decreased life expectancy, with survivorship analysis varying from 87% at 35 years to 71% at 40 years of age, and a mortality rate of 99% by 60 years [24,25,26]. Cor pulmonale and respiratory failure are the most common causes of death [24,27]; hence, diagnosing and treating scoliosis early is of the utmost importance.

The lifetime risk of an individual with PWS developing scoliosis is remarkably high, approximately 70%. Screening should start after the child begins sitting independently and continue on a regular basis until skeletal maturity. Given the lack of vertebral rotation related to the curve, radiographic monitoring is recommended on a yearly basis for children under 4 years of age. The curves seem to result from the characteristic hypotonia, particularly in the younger children. Interestingly, more than half of the curves occur in the adolescent period which is also the most common age group seen in idiopathic scoliosis. In idiopathic adolescent scoliosis, a number of associated chromosome abnormalities have been reported (e.g., 1p36.32, 2q36.1, 8q12, 10q24.31, 17q25.3, and 19p13.3) that may involve genes affecting growth and musculoskeletal development [28]. For example, next-generation sequencing, genotyping, linkage analysis, GWAS and gene expression studies in tissues have identified 23 genes that are related to scoliosis [29,30,31,32,33,34]. These genes code for extracellular matrix proteins, collagen, bone formation, mineralization and metabolism, growth and sex hormones, and homeobox genes required for differentiation of skeletal elements and structural integrity of the vertebrae. Eight of these 23 genes are located in those associated chromosome regions identified to be disturbed in humans with scoliosis (Online Mandelian Inheritance in Man (OMIM), www.omim.org). It is unclear how causative any of these genes are to the spinal deformity in PWS, and none of the identified genes are on chromosome 15, let alone in the PWS region. However, the genetics of scoliosis in PWS has been poorly studied. Interestingly, both *NIPA1* and *NIPA2* genes are located in the 15q11.2 BP1–BP2 region and the encoded proteins are involved as magnesium or cation transporters with bone morphogenetic protein (BMP) production, signaling pathways or BMP receptors, along with transforming growth factors and their receptors thereby influencing cartilage and bone formation (GeneCards.org; OMIM). [35]. One could speculate that disturbances could impact differentiation of myoblasts and osteoblasts, calcium regulation and bone mineralization, maintenance of neuromuscular junctions and formation of cartilage and bone mass in PWS. The multifactorial nature of scoliosis may also indicate that allelic differences on other chromosomes are necessary for development of scoliosis in children with PWS.

## 2. Clinical Observations and Treatment

### 2.1. Clinical Experiences and Treatment for Scoliosis in Prader–Willi Syndrome 

As there are few reports on treating scoliosis in PWS, a major focus of this report is to describe the best treatment strategies for spine deformities in children with PWS based on 20 years of clinical experience. Clinical observations, risk factors, therapeutic approaches and opinions regarding orthopedic care will be divided into subsections in this report.

#### 2.1.1. Physical Therapy

**Figure 1:**
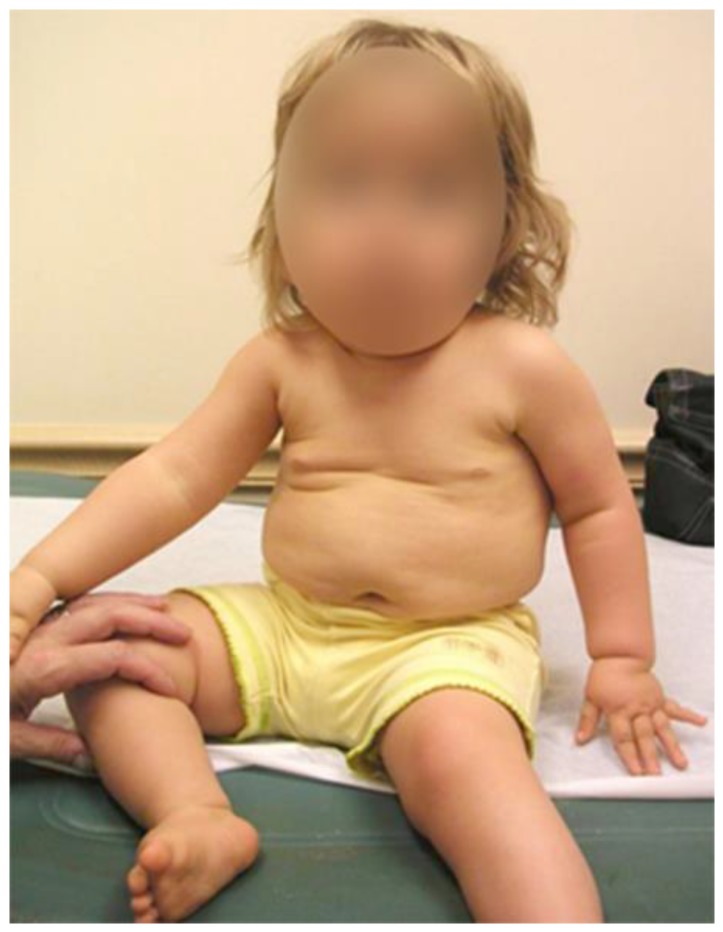
18-month-old female with Prader–Willi syndrome (PWS) maternal disomy of chromosome 15 (UPD) type, displaying the typical hypotonic sitting position.

All children with PWS, except the most atypical, need ongoing therapy services starting as a neonate. Since children with PWS are initially stronger in their extremities rather than their core, emphasis first is on trunk strengthening and sensory integration. Developmental milestones in PWS are delayed with ambulation not occurring until 27 months on average [36]. Although there is a strong temptation to have these children upright, unsupported upright sitting should be delayed until the child has the strength to pull themselves to a sitting position spontaneously. This should prevent initiation of a spine deformity due to a hypotonic slouch when sitting prior to physiological readiness (Figure 1). A reclined seated position (~about 60°) will allow for feeding and appropriate interactions, while preventing postural droop. Similarly, once a child is sitting, emphasis then should be placed on quadruped stance and crawling, while also developing standing skills. Solid ankle-foot-orthoses will stabilize ankle weakness and hyperlaxity. At risk children may also benefit from hippotherapy for spine strengthening and balance at a suitable age.

#### 2.1.2. Screening

Scoliosis in infants and children with PWS is more difficult to diagnose clinically compared to children with idiopathic scoliosis [37]. The spine in patients with PWS exhibits much less rotation relative to curve size, resulting in diminished posterior rib asymmetry. Because the incidence of scoliosis is high in the infantile period in PWS, routine radiographic screening is important for early recognition of children at risk. In the uncommon case of a child showing obvious signs of a spinal asymmetry at an early age, the finding should be appropriately evaluated radiographically. But in the routine case, screening radiographs are first performed once a child can sit unassisted and are done yearly as seated or standing spine films until about 4 years of age. The risk of developing a curve thereafter is relatively small until the child nears adolescence. Any deviation from straight should probably be followed by a physician experienced in treating pediatric spine deformities, so they can verify measurements, and set a schedule for clinical and radiographic follow-up.

#### 2.1.3. Serial Spinal Casting

**Figure 2:**
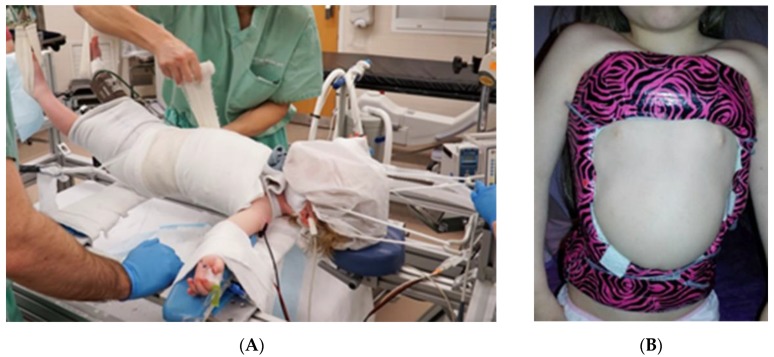
(**A**) Patient on the Risser casting table. Note the straps from the head halter traction, as well as the pelvis traction straps visible lateral to the patients left hip. (**B**) Patient in a spine cast.

Serial casting for spine deformities in infants and young children has had a resurgence of interest after Mehta published her article in 2005 [38] and others [39]. Spine casting is done on a special Risser table, where the child, under general anesthesia, has traction applied through a head halter traction and crisscrossing pelvis straps. The table top can then be lowered, so that the patient is suspended by transverse bars at the level of the shoulders and pelvis, which allows for full circumferential exposure of the trunk and chest. Casting material is applied, and the appropriate maneuver of chest derotation and sagittal translation for curve correction is performed (Figure 2). The cast is changed every 2 months for those younger than 2 years, every 3 months for those between 2 and 3 years of age, and every 4 months for those over 3 years. 

**Figure 3:**
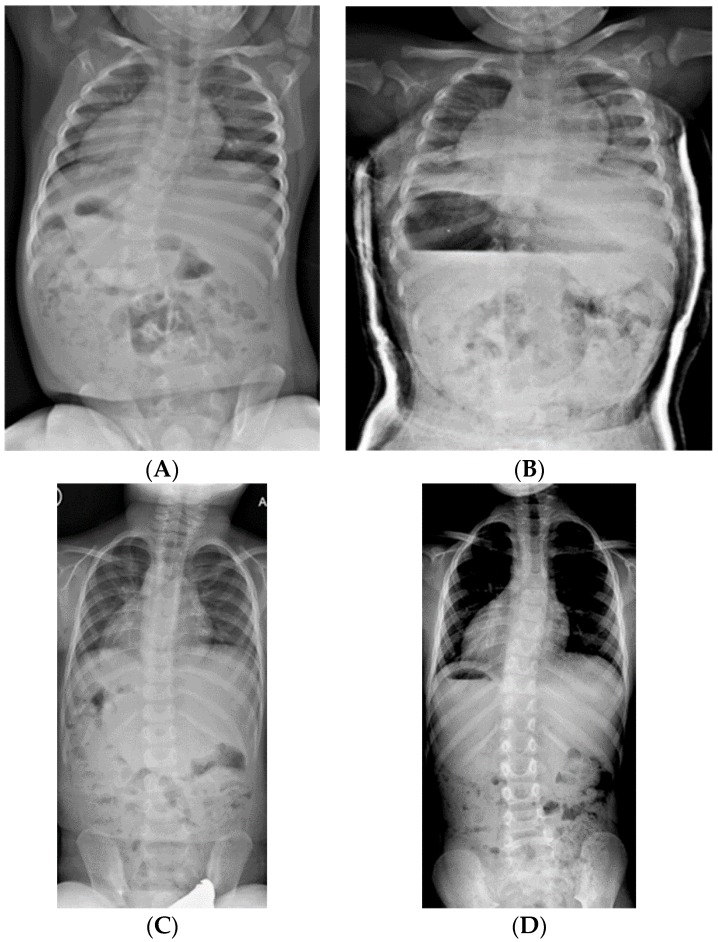
(**A**) 19-month-old male with PWS deletion type with 52° curve. (**B**) Same patient at 20 months old, sitting in his first spinal cast, 10° curve in cast. (**C**) Same patient at 35 months old, after completing 8 casts, 11° curve. (**D**) Same patient at 6 years old, 3 years out of cast and 2 years out of brace, with an 18° curve.

**Figure 4:**
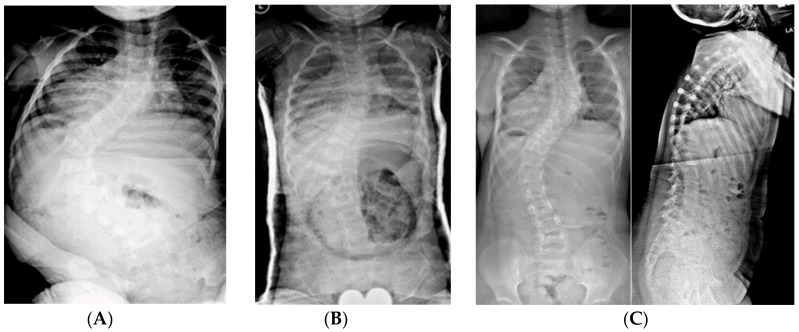

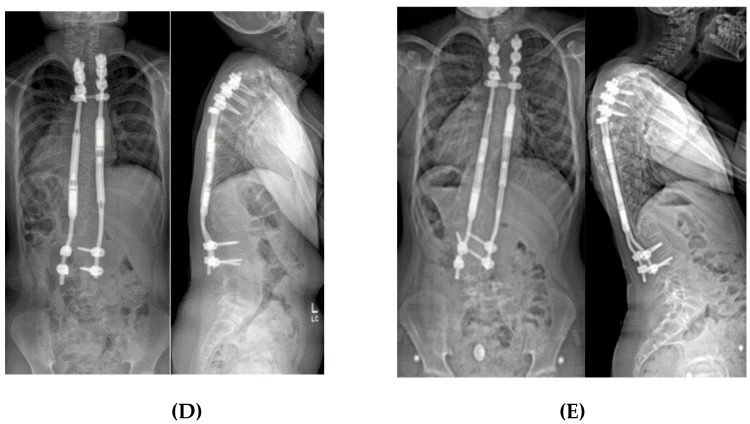
(**A**) 18-month-old female with PWS deletion type with 106° curve. (**B**) Same patient at 18 months old, sitting in first cast, 54° curve. (**C**) AP and lateral views of spine at 6 years old, after 18 casts, 61° curve, just prior to surgery. (**D**) AP and lateral views of spine 4 months after placing magnetically actuated spine rods T3–L3. Curve was corrected to 30°, maintaining her pre-operative kyphosis. (**E**) AP and lateral views of spine at 10 years old, just prior replacement with new expandable rods. Due to adding on, curve measures 50°, which was addressed by including L4 in the construct.

Although there is the occasional case where an infantile curve spontaneously resolves, most curves over 25° require treatment. If a curve would naturally correct without treatment, it will merely correct quicker with treatment. Therefore, treating all curves at 25° appears to improve chances of intervening at the best possible moment to help a curve resolve. Indications for starting spine casting is a curve greater than 25° in a child over sitting age and usually younger than 3 years of age; we have initiated spinal casting on the occasional child at nearly 5 years of age. In the rare case when a child has a large curve before they begin sitting independently, a semi-rigid spine brace is used to temporize until they are suitably developed physically to start casting. There are three possible outcomes from cast treatment. The best outcome (“cured”) is the curve that can be reduced to either <15° out-of-cast, or 3 successive out-of-cast measurements of <25°, in which case the patient is transitioned to a brace, then weaned out of the brace after one year (Figure 3). More moderate curves, once they plateau in casting, are also transitioned to a brace, with the expectation that the brace will be long-term treatment. Severe curves are casted until the child is old enough to undergo surgery for an expandable spine implant (5–8 years of age) (Figure 4). 

Oore et al. reported the only published series of serial casting for children with PWS, noting a reduction of curve size in their ten patients, from 45° to 37° [40]. We reviewed our results from 34 children with PWS with more than 24 months’ follow-up after their initial spine cast. The average age at initial casting was 32 months (range 14–64 months) with an average of 8 casts (range 3–18). Twelve children (35%) were in the “cured” group; all were weaned out of brace at one year after completion of casting. Seven of these patients had maternal disomy 15, five had the 15q11-q13 deletion, and the average initial curve was 44° (range 27°–80°). Another 18 patients transitioned to long-term brace wear (10 with deletion, 7 with maternal disomy 15, and one having an imprinting defect). Their average initial curve of 55° (range 27°–84°) became 35° (16°–64°) at the end of casting and was 46° (27°–84°) at two years’ follow-up. Four patients with severe initial curves (54°, 84°, 95°, and 106°) were controlled in casts until they reached a sufficient age for surgery (average 56 months old). Overall, the odds ratio for “curing” an initial curve of <50° was nine times that of a curve >50° in this cohort.

#### 2.1.4. Bracing

**Figure 5:**
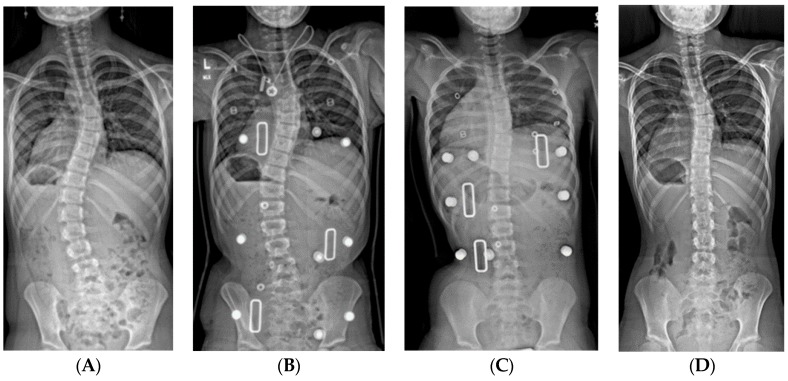
(**A**) 10-year-old female with PWS deletion type and 41° curve. (**B**) Standing in her daytime thoracic-lumbar-sacral orthosis (TLSO). (**C**) Supine in her nighttime Providence brace. (**D**) At 13 years, with curve improved to 25°.

Bracing is most appropriate for children who have spinal curves over 20°–25° but too old for casting. The goal of bracing is to prevent curve progression, but in very large curves, braces may be used to control the curve until the patient reaches a desired age for surgery. In general, curves smaller than 30° are treated with a nighttime only Providence style brace. This brace is designed to obtain maximal curve stretching, maintaining its flexibility. The brace is not appropriate for ambulation. If the curve exceeds 30°, a standard thoracic-lumbar-sacral orthosis (TLSO) is added for daytime use. Our preference is an anterior opening, custom molded brace, with a goal of achieving 40–50% diminution of the curve based on an in-brace spinal radiograph. The use of two braces together may seem excessive, but unanticipated curve improvement has occurred with this strategy (Figure 5). If a curve progresses past 50°, and future surgery is likely, then brace treatment is continued so long as in-brace radiographs show the curve below 50°, allowing the child the opportunity for further growth.

#### 2.1.5. Spine Surgery

##### 2.1.5.1. Complications

Prior to discussing the different surgical options for children with PWS and severe scoliosis, it is important to understand why their scoliosis is different from idiopathic spine deformities, as well as what makes the child with PWS different from the typical child with regards to major surgery. It is important to think of the patient as a child with PWS who has scoliosis, and not as just a child with scoliosis who also has a rare and obscure diagnosis.

**Figure 6:**
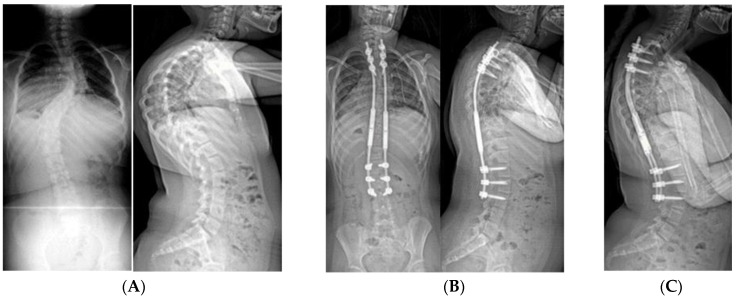
(**A**) 10-year-old female with PWS UPD type with 85° kyphosis, a 66° right thoracic and 61° left thoracolumbar curve. (**B**) Expandable, magnetically actuated rods are implanted from T2–L3, reducing her scoliotic curves to 25° or less, and her kyphosis to 48°. (**C**) 6 months post-operatively with developing proximal junctional kyphosis. Kyphosis now measures 81°.

**Figure 7:**
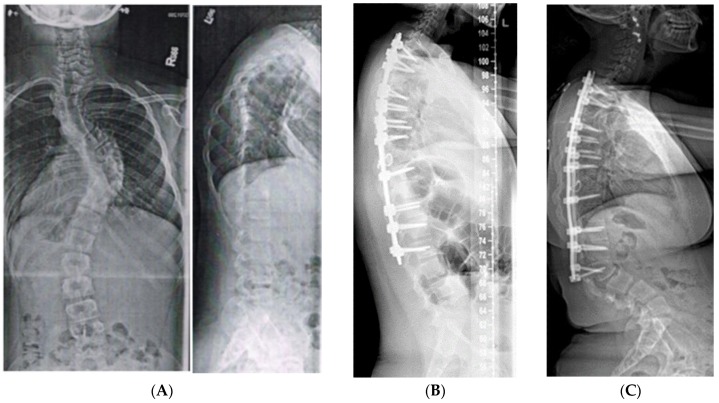
(**A**) 11-year-old female with PWS UPD type with 70° scoliosis, and 60° kyphosis. (**B**) Same patient at 12 years of age, after T2–L2 posterior spinal fusion. Overall kyphosis measures 50°. (**C**) Same patient at 3 years post-operatively with 60° distal junctional kyphosis.

The most common complication affecting outcomes of spinal instrumentation is adding-on of the curve above or below the construct, usually as proximal or distal junctional kyphosis (PJK and DJK, respectively); PJK being the most frequent problem (Figure 6 and Figure 7). Patients with PWS characteristically have a “head forward” posture with their C7 vertebral plumb line falling much farther anterior to the sacrum than would be seen in a typically developed person [11,13]. This occurs by a combination of flattened cervical lordosis and/or thoracic hyperkyphosis. In fact, most PWS curves are kyphoscoliotic rather than the lordoscoloisis seen in idiopathic scoliosis cases. If the sagittal alignment is “anatomically” corrected surgically, patients often will compensate by increasing kyphosis, leading to hypolordosis or even kyphosis in the cervical spine, PJK and DJK [9,41]. In addition, up to 62% of patients with PWS have low bone mineral density and other musculoskeletal manifestations [11,42,43,44,45,46]. The combination of the PJK/DJK and bone weakness leads to a rate of hardware pull out/failure/rod fracture between 17% and 31% [9,40,41,47,48]. The consequences can be catastrophic [49]. 

In surgical planning, positioning and density of anchoring hardware (primarily pedicle screws) should be chosen with osteoporosis/osteopenia in mind. Thoracic kyphosis should be addressed judiciously – patients with kyphosis or kyphoscoliosis should not have their sagittal profile corrected to values appropriate for typical patients. In most kyphotic deformities, we strive for a resulting kyphosis of 50° to 60°, depending on the initial measurement and sagittal balance. We will select T3 or lower as our upper instrumented vertebra, to allow enough room for the patient to adjust their kyphosis across the cervical-thoracic junction.

Patients with PWS exhibit “skin picking”, which can lead to invasion or injury of their incisions. Combined with potential poor quality of the soft tissues, the rate of wound dehiscence and related deep infections are reported between 19% and 38% of cases [40,41,48]. To avoid these issues, a negative pressure wound therapy bandage is placed over the incision for the first five days, both to help with any seepage and to act as an “early warning” system, as the pump will alarm if the patient breaks the pressure seal by scratching their incision. Afterwards, they are outfitted with a light brace to be worn at all times except bathing, primarily to protect the incision for 2–3 months.

Patients with PWS often have gastrointestinal problems and are nearly universally constipated. They also experience a protracted ileus post-operatively which can substantially delay recovery. Their interest in food reemerges soon after waking from surgery. If not monitored, they can easily overeat, hyperextending their stomach, and developing gastroparesis and stomach rupture, a life-threatening complication of PWS. Our protocol is for a slow bowel clean out with stool softeners and laxatives for one to two weeks prior to surgery. Post-operatively, limited clear fluids (2-4 ounces per 4 hours) are allowed until stomach sounds return. Erythromycin is used to increase gastric motility while methylnaltrexone blunts the effect of opioids on the gastrointestinal system and lactulose stimulates bowel movements. The diet is advanced slowly, relative to bowel sounds, and abdominal radiographs are obtained usually daily to monitor for signs of gastric stasis until regular bowel movements are established. 

Intravenous access can be difficult in children with PWS. Since there may be a prolonged need for intravenous hydration, a central venous line is placed in the operating room, to prevent having to search for venous access in the post-operative period.

As noted in the introduction, children with PWS often have sleep apnea or other respiratory conditions; sleep apnea occurs in 80% of children, and narcolepsy in 36% [16]. Pre-operatively, a sleep study is needed along with a pulmonology evaluation to assess for the possible post-operative needs such as the use of BiPAP or CPAP. Occasionally, if they are slow to awake after surgery, they will remain intubated until they demonstrate a strong respiratory response. 

##### 2.1.5.2. Non-Fusion Spinal Instrumentation with Expandable Implants

**Figure 8:**
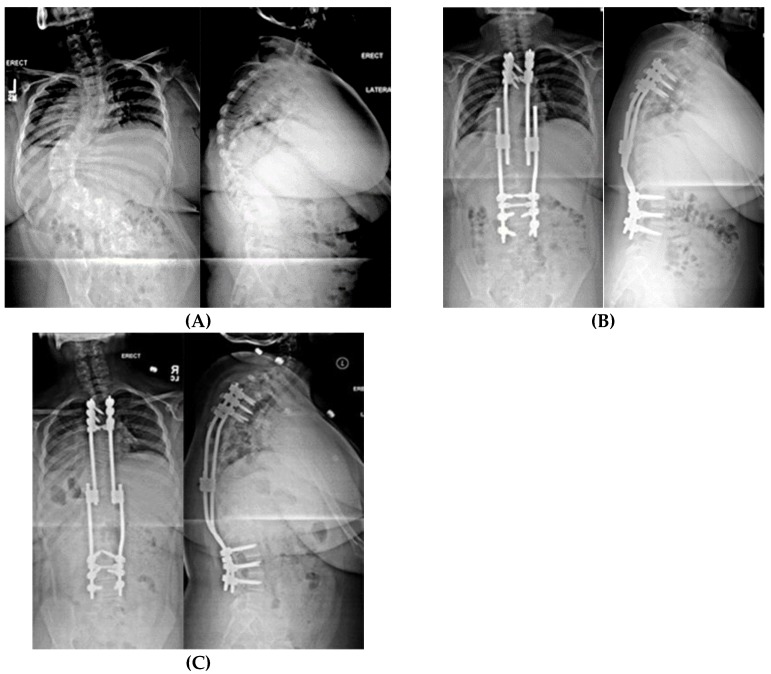
(**A**) AP and lateral radiographs of a 10-year-old female with PWS deletion type with 103° curve. (**B**) AP and lateral radiographs 1 month later, after placement of non-fusion spinal instrumentation. Curve is 57°. (**C**) 18 months later, after 50mm of lengthening, curve is 25°.

Young children with severe infantile curves may need stabilization of the spine to allow for more symmetric chest development with growth. Typically, these are child too old for spinal casting but have substantial remaining growth with curves >50° and minimal flexibility/improvement in brace. If they cannot be maintained in brace, then an expandable implant serves to internally splint the spine. Early implant versions required manual distraction, typically performed every six months as an open procedure in the operating room (Figure 8). This strategy worked well for controlling the curve and maintaining spine growth, but with the potential complications related to surgical procedures. In 2014, expandable spinal rods became available that were magnetically actuated through the skin. The rods are lengthened every three months, using an external spinning magnet, which causes the magnet within the rod to rotate thereby lengthening the rod (Figure 4). Oore et al found that the average initial curve was reduced from 76° to 42° at time of implant surgery with no change in curve size over the subsequent two years of rod elongation [40].

Expandable implants, as a technique, have a life span of approximately five years, after which the ongoing process of spontaneous fusion along the section of the instrumented spine leads to stiffness and an inability to lengthen further [50,51]. Therefore, the goal is to manage the curve non-operatively until the child is 5–8 years of age, in order to “grow” the spine until at least 10 years of age. Unfortunately, the magnetically actuated rods only generate 42 pounds of axial force and may fail to generate enough force to elongate after several lengthening procedures. There are also issues related to titanium wear debris noted at the time of device removal [52]. Generally, these devices have made important improvements in the treatment of severe curves seen in young children with PWS. Usually, the construct is anchored with two vertebral levels above and below, but when there is any question of bone strength, three levels are fixated.

##### 2.1.5.3. Spinal Fusion

**Figure 9:**
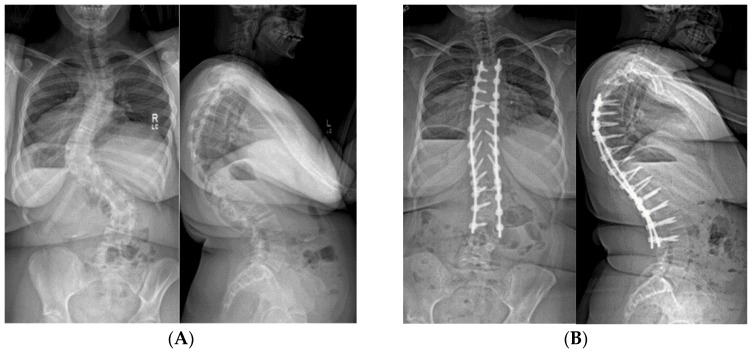
(**A**) 15-year-old female with PWS UPD type with 67° left thoracic and 60° right lumbar curve. She had thoracic kyphosis of 79° and lumbar lordosis of 84°. (**B**) Same patient at 4 years after T4–L3 posterior spinal fusion. The thoracic curve measures 12°, the lumbar 25° with 75° kyphosis.

Spinal curves over 50° have a high propensity towards progression, even in skeletally mature patients; curves over 40° need to be closely monitored for progression. Timing of surgery can be tricky, adolescents with PWS experience delayed puberty, and females may not reach menarche or begin menses until in their 20s. PWS specific growth charts do indicate that boys and girls reach skeletal maturity essentially at the same age as their typically developing peers [53]. It is, therefore, preferable to delay spinal fusion until or after 12 years of age for females and 14 years for males. In general, teenagers with PWS have some concept of body image, but seem less perturbed by residual shoulder asymmetry or waist clefts. Achieving appropriate sagittal alignment is much more important in avoiding late deformity and assuring patient satisfaction. We try to limit our upper end vertebra to T3 or lower, even if still within the region of kyphosis; the rods are bent to accentuate kyphosis. Distally, the end vertebra is chosen much as it is for idiopathic scoliosis cases with a predisposition to fuse a level longer rather than shorter to prevent adding-on of the curve. The rods are bent to match or accentuate the pre-operative lumbar lordosis. Due to concerns of bone strength, nearly all included vertebrae are instrumented with pedicle screws bilaterally for better fixation (Figure 9).

## 3. Discussion

Growth hormone (GH) therapy was approved by the FDA in 2000 for treating genetically confirmed individuals with PWS with short stature. Growth hormone acts as a ligand for the growth hormone receptor (GHR). Approximately 50% of Caucasians with or without PWS and regardless of PWS genetic subtype have a *GHR* gene (exon-3 deletion) polymorphism which is associated with an increased response to growth hormone therapy with growth acceleration [54,55,56]. A significant increase in growth rate (1.7 times) is observed in both PWS and the general population when the *GHR* gene polymorphism is present. In a case-controlled study of 73 patients with genetically confirmed PWS matched for levels of scoliosis (moderate or severe) and those without scoliosis, no differences were found between the two groups in terms of gender, PWS genetic subtype or GHR gene exon-3 deletion distributions [56]. Therefore, the growth rate stimulation by the *GHR* gene polymorphism did not appear to influence the development of spine deformities in PWS.

When treated with GH, individuals with PWS respond favorably in stature, lean body mass and physical activity [1,4]. A frequent concern of parents of patients with scoliosis is whether the growth hormone could be contributing to their child’s curve progression, and therefore whether the hormone supplementation should be stopped. This concern stems from similar apprehension related to the treatment of Turner syndrome, where a higher prevalence of scoliosis in patients treated with GH was suggested in some studies, but refuted by others [57,58]. Studies of children with PWS have been less equivocal. For example, de Lind van Wijngaarden et al. conducted a prospective randomized, controlled GH study of infants, prepubertal and pubertal children [59]. They found a similar onset and progression of scoliosis between those treated with GH and those not, across all three age groups. Nakamura et al. had similar findings in a retrospective study, but also noted that the GH-treated patients had improved bone mineral density as measured by dual-energy x-ray absorptiometry [60]. Other studies of children with PWS and scoliosis have noted significant impacts of GH on metabolism, lean body mass, brain development and energy level, supporting their recommendations to not discontinue treatment [4,42,60]. Data analysis of the 2007 PWSA (USA) survey found that for every month delay in beginning GH treatment, the risk of needing scoliosis surgery increased by 0.7%. Our protocol is to continue GH treatment in all cases, unless contraindicated by the patient’s endocrinologist. At the time of surgery, we will only skip the dose the day of the procedure, resuming on the first post-operative day.

Other than the similarity of age of onset of the adolescent form and the overall coronal plane curve appearance, there are a number of important differences between idiopathic scoliosis and the PWS type. The most obvious is that idiopathic deformity is a lordoscoliosis of the thoracic region, while in PWS most curves are kyphoscoliotic. The exaggerated kyphosis seems to be requisite for proper overall center of balance, as correcting it surgically leads to the greatest post-surgical complications – proximal and distal junction kyphosis. Studies are needed of skeletally mature individuals with PWS, without scoliosis, to determine their natural sagittal posture, which can then inform the goals of the post-surgical alignment. Although neurological and genetic factors do play a role in scoliosis [28], no genes as yet are identified or associated with scoliosis in children with PWS, but future research is needed. For example, expression patterns for both coding and non-coding genes from tissue (e.g., skeletal muscle, bone, connective tissue) and related gene variants may prove as useful markers for development of scoliosis at a young age requiring careful surveillance. 

Experience has taught us that aggressive non-surgical treatment can be successful in postponing or avoiding surgery, and even reducing the actual curve. One-third of the patients that underwent spine casting for their infantile curves had their curve decreased to the point that they could be transitioned to a brace for a year, then weaned from the brace. This is a smaller proportion of “cured” patients compared to Mehta’s series (69%) [38], although the average initial curve of her 136 patients was 38°, compared to 55° for our cohort. Likewise, in brace treatment, at least two older patients had significantly improved curves with separate nighttime Providence and daytime TLSO braces (Figure 5). This most likely indicated a gradual improvement of the patients’ hypotonia while maintaining their spine flexibility and applying corrective external posturing. The strengthening spinal muscles over time were able to preserve the alignment. Naturally, all of these patients need to be carefully followed even after growth cessation to verify no deterioration over time.

It cannot be overemphasized that patients with PWS treated for scoliosis should have their underlying condition understood, particularly when planning surgery. By taking into account such characteristics as hyperphagia, skin picking, the occasional violent outbursts, and planning accordingly, the patient, family and hospital staff can be kept safe during the inpatient stay. Moreover, slow advancement of diet with diligent attention to gastrointestinal function will actually decrease the length of hospital stay; if the diet is advanced too quickly, the subsequent bloating and ileus resumption may take days to resolve.
